# Approach for Detecting Attacks on IoT Networks Based on Ensemble Feature Selection and Deep Learning Models

**DOI:** 10.3390/s23177342

**Published:** 2023-08-23

**Authors:** Shaza Dawood Ahmed Rihan, Mohammed Anbar, Basim Ahmad Alabsi

**Affiliations:** 1 Applied College, Najran University, King Abdulaziz Street, Najran P.O. Box 1988, Saudi Arabia; sdrihan@nu.edu.sa (S.D.A.R.);; 2 National Advanced IPv6 (NAv6) Centre, Universiti Sains Malaysia (USM), Gelugor 11800, Penang, Malaysia

**Keywords:** Internet of Things, deep learning models, ensemble feature selection, IoT attacks, Recursive Feature Elimination (RFE), IoT-Botnet 2020 dataset

## Abstract

The Internet of Things (IoT) has transformed our interaction with technology and introduced security challenges. The growing number of IoT attacks poses a significant threat to organizations and individuals. This paper proposes an approach for detecting attacks on IoT networks using ensemble feature selection and deep learning models. Ensemble feature selection combines filter techniques such as variance threshold, mutual information, Chi-square, ANOVA, and L1-based methods. By leveraging the strengths of each technique, the ensemble is formed by the union of selected features. However, this union operation may overlook redundancy and irrelevance, potentially leading to a larger feature set. To address this, a wrapper algorithm called Recursive Feature Elimination (RFE) is applied to refine the feature selection. The impact of the selected feature set on the performance of Deep Learning (DL) models (CNN, RNN, GRU, and LSTM) is evaluated using the IoT-Botnet 2020 dataset, considering detection accuracy, precision, recall, F1-measure, and False Positive Rate (FPR). All DL models achieved the highest detection accuracy, precision, recall, and F1 measure values, ranging from 97.05% to 97.87%, 96.99% to 97.95%, 99.80% to 99.95%, and 98.45% to 98.87%, respectively.

## 1. Introduction

The Internet of Things (IoT) has emerged as a transformative innovation, enabling various everyday products and gadgets to connect to the web and exchange data. This technology can potentially revolutionize several industries, fostering increased efficiency, improved decision-making, and the development of novel services [1]. However, along with its advantages, the rapid expansion of IoT has led to growing security concerns. The surge in IoT attacks poses a significant threat to organizations and individuals, making it crucial to develop robust intrusion detection systems (IDS) to safeguard IoT networks [2].

The complexity of IoT networks, with their diverse devices and communication protocols, presents unique challenges for traditional IDS. Conventional IDS may struggle to recognize novel or complex attack patterns due to a lack of knowledge about IoT devices and their vulnerabilities. Additionally, the encrypted and encapsulated communication in IoT gadgets further complicates detection. Moreover, the limited processing power of IoT devices inhibits the deployment of standard IDS agents on these devices [3]. Furthermore, IDS’s ability meant to detect IoT attacks relies on inappropriate features that do not contribute to detecting IoT attacks, degrading its detection accuracy. In this research, we address the challenge of detecting attacks on IoT networks by proposing an approach that combines ensemble feature selection with deep learning models.

Our proposed approach comprises two primary stages. Firstly, we employ an ensemble feature selection mechanism that combines various filter techniques to identify the most relevant and discriminative features from the vast IoT data pool. This ensemble feature selection aims to enhance attack detection accuracy by providing deep learning models with more informative inputs. Secondly, we design, develop, and train deep learning models, including Convolutional Neural Networks (CNNs) and Recurrent Neural Networks (RNNs), based on the features selected by the ensemble mechanism. Deep learning models have shown significant advantages in capturing complex patterns and representations from raw data, eliminating the need for manual feature engineering. This enables our models to effectively analyze sequential and temporal data often encountered in IoT network traffic analysis [4,5,6]. The contribution of this paper is as follows:

- An ensemble feature selection mechanism is employed to identify significant features that play a crucial role in detecting IoT-based attacks. The proposed ensemble feature selection mechanism aims to enhance attack detection accuracy.
- DL models are designed, developed, and trained based on the features selected by the proposed ensemble feature selection mechanism to detect IoT attacks. This approach ensures that the DL models focus on the most informative features, leading to enhanced performance in attack detection.
- Comprehensive evaluation of various DL models to evaluate the impact of the proposed ensemble feature mechanism on the performance of DL models.

The remainder of the paper is laid out as follows: In Section 2, we provide the background of this research. Section 3 discusses related works. The approach is described in detail in Section 4. The experimental findings are presented in Section 5. Conclusions and Future Directions are discussed in Section 6.

## 2. Background

This section introduces the IoT attack concerns and feature selection algorithms, as shown in Section 2.1 and Section 2.2, respectively.

### 2.1. Internet of Things Attacks

The proliferation of connected devices and services has significantly improved productivity and ease of life. However, it has also prompted worries about safety. Cyberattacks targeting IoT devices, such as smart homes, wearables, and industrial sensors, are common because of their openness and connectivity targets. The loss of confidentiality, authenticity, and safety are all possible outcomes of such attacks. Users and businesses must be familiar with the many forms of IoT attacks [7].

Distributed denial-of-service attacks, or DDoS attacks [8,9], are a prevalent kind of IoT attack. An army of hacked computers, or “botnets”, is used to overwhelm the network of an intended victim. The targeted device or network becomes unreachable to authorized users due to the overwhelming volume of data. DDoS attacks on connected devices have been very harmful in recent years in several high-profile instances, such as the Mirai botnet attack in 2016 [10].

Data breaches are another common kind of IoT attack. Due to the large volumes of sensitive information that IoT devices collect and communicate, they are being targeted by hackers. By penetrating weak points in IoT security procedures, hackers may steal sensitive information such as credit card numbers, medical records, and even private videos. Identity theft, extortion, or bodily injury caused by hacked equipment like home security systems are all possible results of such breaches.

Additionally, device hijacking is a major issue in IoT security. An adversary compromises an IoT device, taking over its capabilities and influencing its behavior for evil ends. One such example is the manipulation of industrial processes or the hijacking of smart home devices. Attacks like this are dangerous because they threaten national security, public safety, and the privacy of individuals.

Several safeguards may be set up to prevent attacks via the IoT. Users should ensure they have changed their IoT devices’ default usernames and passwords. In addition to doing regular firmware upgrades, manufacturers should also offer patches to address known vulnerabilities. In addition, by isolating IoT gadgets from mission-critical infrastructure, network segmentation might lessen the severity of attacks. Finally, security measures and encryption should be prioritized to protect sensitive information during transmission across networks [11]. Table 1 shows the comparison between IoT Attacks and traditional Attacks.

In conclusion, with the increasing number of interconnected devices, the risk of IoT attacks rises significantly. Understanding the various attacks to protect our information and devices from harm is crucial. By developing a secure and resilient IoT ecosystem, we can mitigate the risks associated with these attacks, enabling individuals and businesses to leverage the benefits of IoT while safeguarding themselves. Proactively addressing IoT security challenges can ensure a safer and more reliable IoT landscape for everyone involved.

### 2.2. Feature Selection

Choosing the most useful features from a dataset is a crucial step in machine learning, which is why feature selection is important. It’s useful for boosting model efficiency, simplifying calculations, and adding clarity to results. Filter and wrapper techniques are two common feature selection techniques [12].

Filter feature selection techniques consider the features’ underlying attributes and statistical statistics to determine the essential features. These techniques analyze the correlation between each feature and the outcome variable in the machine learning technique of interest. The information gain, chi-square, and correlation-based feature selection filters are all examples of such strategies. Filter methods are effective in dealing with high-dimensional data and are computationally efficient. However, they risk missing out on important subtleties due to oversimplification. On the other hand, feature subsets in a wrapper are evaluated based on how they affect the efficiency of a certain machine-learning method. Wrapper approaches use cross-validation and different assessment strategies to determine the quality of feature subsets by using a particular learning algorithm (such as Decision Trees (DT) or Recursive Feature Elimination (REF)). The interaction between features and the selected algorithm is considered by wrapper techniques, which might result in a more precise feature selection. Yet, they may be resource-heavy on the computer, particularly when applied to massive datasets [12].

Various techniques, such as the Variance Threshold, Mutual Information, Chi-square, Analysis of Variance (ANOVA), L1-based, and Recursive Feature Elimination (REF), are used for feature selection. A brief description of these techniques is as follows:

- The Variance Threshold (VT) [13] technique discards information with low variance on the premise that these characteristics are less useful for making accurate predictions.
- Mutual Information (MI) [14] is a statistical indicator of the degree of correlation between characteristics and the dependent variable of interest. A higher level of mutual information between two features indicates greater importance.
- The chi-square (Chi) [15] test is a technique for assessing the association between a set of categorical predictors and an equally categorical outcome variable. As a result, we may zero in on the characteristics most likely to be linked to the dependent variable.
- Analysis of Variance (ANOVA) [16] evaluates the statistical significance of a numerical feature’s association with a categorical target variable. Features with a strong influence on the dependent variable are isolated.
- L1-based [17] penalizes features with tiny coefficients using regularization strategies such as Lasso. Some feature coefficients are adjusted to zero to promote sparsity in feature selection.
- Recursive Feature Elimination (REF) [18] trains and evaluates models on multiple feature subsets to reduce less significant features iteratively. As the model’s performance is evaluated, characteristics are eliminated one by one.

Statistical characteristics, information theory, and model-based tactics all play a role in these approaches to feature selection. The trade-off between model accuracy and complexity is one consideration, but the properties of the data, the nature of the issue, and the approach used all play a role. Using the right feature selection strategies may boost our models’ effectiveness, interpretability, and generalizability.

## 3. Related Works

E-Spion [19] is an IDS for IoT devices that profiles their behavior using system-level data to identify abnormal intrusion behaviors. E-Spion’s three layers of security enhance detection efficacy but increase the devices’ operating expenses. E-Spion’s performance is evaluated using a dataset of 3973 IoT malware samples on a testbed. Detection efficiency ranges between 78% and 100% based on the number of detection layers employed. They evaluated the various E-Spion layers’ ability to detect anomalies and cost overhead.

Shareena et al. [20] proposed a deep learning-based IoT DDoS botnet intrusion detection system. To effectively identify IoT botnet attacks, they designed a highly extendable Deep Neural Network (DNN) using data collected from a realistic network environment. The results demonstrated that the proposed DNN identified IoT DDoS botnet attacks with higher levels of precision and accuracy than existing systems.

Nimbalkar et al. [21] proposed an approach for feature selection in IDSs that seeks to recognize DoS and DDoS attacks. Their approach included insertion and union operations on feature subsets containing the top 50% IG and GR values. In tests on the IoT-BoT and KDD Cup 1999 datasets using a JRip classifier, their approach was superior to the original feature set and conventional IDSs while still needing just 16 and 19 characteristics, respectively.

An ML-based IDS method, implemented by Li et al. [22], uses ensemble trees of DT and RF classifiers. Their approach attempted to boost attack detection efficiency by providing information for the ML model’s inferences. They used the NF-BoT-IoT-v2, NF-ToN-IoT-v2, and IoTDS20 datasets to evaluate the approach using the net flow meter feature set.

To create an effective and trustworthy IIDS, Priya et al. [23] proposed training a Deep Neural Network (DNN) to detect and foresee intrusions on networks of the IoMT (Internet of Medical Things). Improved accuracy and a 32% decrease in calculation time were both obtained by the proposed DNN framework, allowing for faster detection to reduce post-intrusion impacts in essential cloud computing. As the IoT and the Internet economy continue to expand at a fast pace, network security becomes even more critical.

The need for reliable and trustworthy data for effective IoT applications was highlighted in a recent paper by Sriram et al. [24], who proposed a deep learning approach for botnet detection based on network traffic flow. Using Long Short-Term Memory Networks (LSTM) autoencoders and CNN, Yin et al. [25] created a deep learning model for anomaly detection in IoT networks. Although their method only applied to binary classification, they used a two-stage window-based data preparation strategy to enhance learning predictions. The F1 score, accuracy, precision, and recall all improved.

Using CNN-based anomaly-based IDS, Saba et al. [26] created a deep learning-based strategy to improve IoT security. Their method evaluated the entire traffic throughout the IoT network to identify potential intrusions and aberrant traffic behavior. Using the BoT-IoT datasets, they found that their technique had an accuracy of 92.85%.

Wang et al. [27] built a deep hierarchical network that can learn traffic characteristics from raw packet data to analyze malicious activity at the packet level. Features in both space and time were retrieved using a CNN and a Gated Recurrent Unit (GRU). In their model, specific attacks were easily detected, while others were not.

In the context of medical devices, Manimurugan et al. [28] proposed a DL-based approach to intrusion detection in IoT systems. The proposed approach detects many attacks and anomalies with high precision. The proposed method showed high accuracy across different classes, with 99.37% accuracy for the normal class, 97.37% accuracy for the Botnet class, 97.71% accuracy for the Brute Force class, 96.67% accuracy for the Dos/DDoS class, 96.37% accuracy for the Infiltration class, 9771% accuracy for the Ports can class, and 98.37% accuracy for the Web attack class.

The paper proposes an efficient anomaly detection mechanism for an IoT network using mutual information (MI) and a deep neural network (DNN). The proposed model improves accuracy and reduces the false alarm rate compared with other deep learning models.

For feature selection and anomaly detection in the IoT for smart cities, Li et al. [29] proposed a model architecture for deep migration learning that integrates deep learning with intrusion detection. In their research, the authors present a method for analyzing learning models for migration and selecting pertinent system features. The KDD CUP 99 dataset was chosen as the experimental dataset, and 10% of the data was utilized as training data. Experimental results show that the proposed algorithm outperforms the existing algorithms when compared with the proposed algorithm.

The work [30] proposed an efficient IoT network anomaly detection mechanism using mutual information (MI) and a DNN. Compared to other deep learning models, the DNN-based NIDS model achieves higher accuracy and reduces false alarms. The study uses the publicly available IoT-Botnet 2020 dataset, which contains 85 features of various data types. By selecting the top features through MI, the model’s accuracy improves by 0.57–2.6%, and false alarms decrease by 0.23–7.98%. Table 2 shows a summary of existing research.

Overall, researchers have explored various deep learning-based approaches to enhance intrusion detection in IoT systems. These studies have improved accuracy, reduced false alarms, and facilitated more effective recognition and detection of IoT attacks. However, the challenge of proposing an efficient feature selection mechanism remains. Existing approaches often rely on a feature selection mechanism that fails to identify the significant features contributing to the detection of IoT attacks. They may select features that could introduce patterns (e.g., source IP address and timestamp), misleading the DL classifier and degrading its performance. By carefully selecting features, evaluating different algorithms, and harnessing the power of deep learning, significant advancements are being made to strengthen IoT security and protect against IoT attacks.

## 4. Proposed Approach

**Figure 1:**
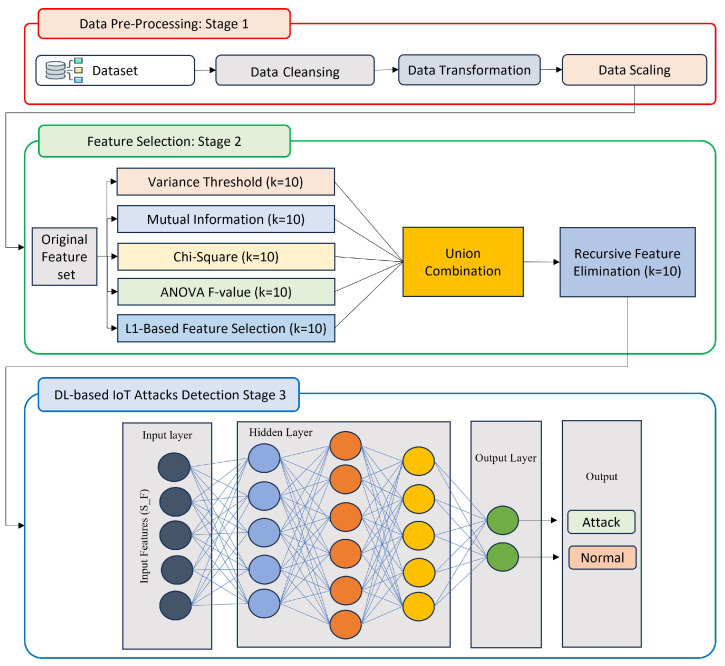
Proposed approach.

This section describes an approach to detect attacks on IoT networks using ensemble feature selection. Figure 1 depicts the proposed approach’s main stages: (1) data pre-processing, (2) ensemble feature selection, and (3) DL-based IoT attack detection. These three phases are discussed in detail in the following subsection.

### 4.1. Data Pre-Processing

Data pre-processing plays a vital role in data science and machine learning, transforming raw data into a suitable format for analysis. This critical phase significantly impacts the quality and effectiveness of machine learning results. In the context of this research, where the effectiveness of feature sets on DL models is being examined, data pre-processing is performed as a preliminary step to prepare the dataset for the DL models. Furthermore, data pre-processing is vital to improving data quality and aiding effective decision-making based on the analysis performed. Several techniques can be involved in data pre-processing. In this research, the dataset used to evaluate the proposed approach has undergone three pre-processing techniques: data cleansing, transformation, and normalization. Data cleansing removes or corrects any errors, inconsistencies, or outliers in the dataset. This helps to ensure that the data is accurate and reliable for analysis. Transformation involves converting the data into a suitable format or scale, such as logarithmic transformation or standardization, to make it more suitable for the DL models. Data scaling is another crucial technique used to scale the data within a specific range, such as between 0 and 1, to ensure that all variables have equal importance in the analysis.

### 4.2. Ensemble Feature Selection

This stage proposes an ensemble feature selection approach to identify the most relevant features contributing to detecting IoT attacks. Relying solely on a single feature selection algorithm can lead to an inappropriate selection of features. Therefore, the proposed approach employs filter, union operation, and wrapper feature selection techniques to select the top 10 best features. Five commonly used filter feature selection algorithms in IDS are utilized to identify the top 10 best features. The filter feature selections used are (I) VT, (II) MI, (III) Chi, (V) ANOVA, and (IV) L1-based. By incorporating multiple algorithms, the ensemble process leverages the strengths of each algorithm [31]. The ensemble is formed by performing a union operation on the outputs of each filter feature selection technique. The primary objective of the union operation is to choose unique features from different subsets. However, this combination method does not consider features’ internal redundancy or irrelevancy in terms of prediction information. It may result in an increased number of selected features [32], but the maximum limit is set to 50 features. To address this, the output of the union operation serves as input to a wrapper algorithm called RFE, which helps eliminate redundancy and retain relevant features. The REF algorithm aims to obtain a highly relevant subset of uncorrelated features, leading to a significant reduction in the dimensionality of the dataset and improved performance of learning algorithms.

It is worth noting that before the ensemble feature selection stage, the features that serve as patterns or are used to construct network flow, such as the source IP, destination IP, source port, destination port, and flow ID used to build network flows, have been excluded from the input feature set. This ensures that these features, which may potentially mislead the deep learning classifiers, do not influence the DL detection process. By removing these pattern features, the ensemble feature selection stage focuses on identifying other relevant features contributing to effective intrusion detection in IoT systems.

While ensemble techniques tend to enhance feature selection performance, we acknowledge the possibility of increased computational overhead due to the combination of these techniques. However, it is worth noting that the feature selection process is performed offline during the model training phase. As such, the computational cost incurred during feature selection is a one-time overhead and does not impact real-time inference when detecting attacks on IoT networks. Moreover, to address any potential issues with redundancy and irrelevance introduced by the union of selected features, we utilize a wrapper algorithm called FE. RFE iteratively removes less important features, refining the feature set and potentially reducing the computational complexity during the training phase of our deep learning models.

The mathematical notations for the ensemble feature selection are as follows:

Let the input feature set be

$$f=\{f_{1},f_{2},f_{3},\ldots ,f_{n}\},$$

where *n* is the number of used dataset features (excluding the label class).

Let the excluded feature set be

$$f_{e}=\left\{f_{e1},f_{e2},\ldots ,f_{ek}\right\}.$$

The input feature set of ensemble feature selection is obtained as:

$$F_{f}=f_{e}-f$$

Define the following feature selection methods:

$$\begin{array}{c} F_{vt}=\mathrm{VarianceThreshold}\left(F_{f},k=10\right)\\ F_{mi}=\mathrm{MutualInformation}\left(F_{f},k=10\right)\\ F_{cs}=\mathrm{ChiSquare}\left(F_{f},k=10\right)\\ F_{an}=\mathrm{ANOVA}\left(F_{f},k=10\right)\\ F_{l1}=\mathrm{L1Based}\left(F_{f},k=10\right) \end{array}$$

The union of these feature sets is denoted as follows:

$$F_{u}=F_{vt}\cup F_{mi}\cup F_{cs}\cup F_{an}\cup F_{l1}.$$

where the number of features of $F_{u}$ is ≤ 50.

The top 10 feature set obtained by REF is represented as follows:

$$F_{REF}=\mathrm{REF}\left(F_{u},k=10\right).$$

The feature set $F_{REF}$ plays a crucial role in detecting IoT attacks and is an essential input for DL models.

### 4.3. DL-Based Model for IoT Attacks Detection

DL models have shown promising performance in detecting and classifying attacks in obscurity and other domains. In this stage, we employ several DL models: CNN, RNN, GRU, and LSTM, as binary classifiers to evaluate the effectiveness of $F_{REF}$ on the performance of the DL model. The DL models used in this research have been selected as they are commonly used in existing research, such as in [33,34]. The dataset is divided using the 80/20 rule [35], known as Pareto theory. Initially, the dataset is stratified to allocate 80% for training and 20% for testing. The training data is generated based on the selected feature set $F_{REF}$ and is utilized to detect IoT attacks in the testing samples. A DL-based model for IoT attack detection is outlined in Algorithm 1.

The step-by-step explanation of Algorithm 1 is as follows:

- The algorithm takes input data, including training, testing, and validation sets (X_train, X_test, X_val, y_test, y_train, y_val), and the selected features (F_REF) as input.
- The DL model architecture is defined, specifying the activation function, batch normalization, dropout layers, and regularization techniques to design the model.
- The DL model is compiled with an appropriate loss function and optimizer.
- The DL model is trained using the training data (X_train, y_train) and validated using the validation data (X_val, y_val). The training continues until the stopping criteria are met, ensuring the model converges and preventing overfitting.
- The algorithm logs the performance metrics and loss function during training to monitor the model’s progress.
- The training process is repeated for a predetermined number of epochs (*n*).
- After training is complete, the algorithm evaluates the performance of the trained DL model using the test data (X_test). It calculates various performance metrics based on the actual labels (y_test) and predicted labels ($y{}^{\prime }$).

**Algorithm 1** DL-based model for IoT attacks detection**Input:** X_train, X_test, X_val, y_test, y_train, y_val, and F_REF (features)**Output:** Performance evaluation metrics**Define DL model (e.g., LSTM) architecture:** Set the activation function, batch normalization, dropout layers, and regularization to design the Deep Learning (DL) model.Compile the DL model using an appropriate loss function and optimizer.**Fit the DL model**: Set the batch size, optimizer, learning rate, number of epochs (*n*), and early stopping criteria (e.g., monitor = ‘loss’ and patience = 3).**for** *i* **in** 1 to *n* **do**   **while** stopping criteria are not met **do**     Train the DL model based on the selected features (F_REF) using X_train, y_train, X_val, and y_val.     Log the performance metrics and loss function during the training process.     **Monitor the loss function** to check for convergence and early stopping.   **end while****end for****Evaluate** the trained DL model using X_test and calculate performance based on the y_test and y_train.

The DL models utilized in this study may vary in their computational requirements. However, we intended to showcase the detection performance of various deep learning architectures. We acknowledge that the choice of the final model may depend on specific use cases, hardware constraints, and desired trade-offs between detection accuracy and computational complexity.

## 5. Experimental Results

This section explains the dataset used to evaluate the proposed approach, discusses the evaluation metrics, reports the results of the proposed ensemble feature selection, provides the results of the DL models, and provides an in-depth discussion of the reported results. These aspects are covered in Section 5.1, Section 5.2, Section 5.3, Section 5.4, and Section 5.5, respectively.

### 5.1. Dataset

In this research, we used the publicly available dataset IoT-Botnet 2020 [36] to evaluate the performance of our proposed approach. This dataset is given in Comma Separated Value (CSV) format and was constructed by parsing the PCAP files of the BoT-IoT dataset [37]. It has more streaming and network characteristics that are important to our research. DoS, DDoS, reconnaissance, and information theft attacks are examples of attacks in the IoT-Botnet 2020 dataset. Table 3 shows the record distribution of the IoT-Botnet 2020 dataset.

Furthermore, the IoT-Botnet 2020 dataset is a flow-based dataset comprising 85 features. This large number of features poses a challenge and necessitates implementing feature selection techniques. Feature selection becomes crucial to identify the most relevant and informative features within the dataset, thereby reducing dimensionality and improving the overall performance of the analysis.

### 5.2. Evaluation Metrics

Various evaluation metrics are employed to evaluate the effectiveness of the proposed approach in detecting IoT attacks. These metrics include precision, recall, detection accuracy, and F1-Measure. They are calculated using the following equations:

(1) $$\mathrm{Precision}=\frac{\mathrm{TP}}{\mathrm{TP}+\mathrm{FP}}$$

(2) $$\mathrm{Recall}=\mathrm{Detection}\;\mathrm{Rate}=\frac{\mathrm{TP}}{\mathrm{TP}+\mathrm{FN}}$$

(3) $$\mathrm{False}\;\mathrm{Postive}\;\mathrm{Rate}=\frac{\mathrm{FP}}{\mathrm{TN}+\mathrm{FP}}$$

(4) $$\mathrm{True}\;\mathrm{Negative}\;\mathrm{Rate}=\frac{\mathrm{TN}}{\mathrm{TN}+\mathrm{FP}}$$

(5) $$\mathrm{Accuracy}=\frac{\mathrm{TP}+\mathrm{TN}}{\mathrm{TP}+\mathrm{TN}+\mathrm{FP}+\mathrm{FN}}$$

(6) $$F1\;\mathrm{Measure}=2\times (\frac{\mathrm{Precision}\times \mathrm{Recall}}{\mathrm{Precision}+\mathrm{Recall}})$$

The evaluation metrics used in this study are widely recognized as standard metrics for assessing the effectiveness of IDS. They provide valuable insights into the performance of the proposed approach in detecting IoT attacks. Moreover, these metrics have been extensively utilized in previous research studies, reinforcing their significance in IDS evaluation, such as in ref. [34,38,39,40,41].

### 5.3. The Result of Ensemble Feature Selection

This section presents the results of the filter feature selection methods (VT, MI, Chi, ANOVA, and L1-based) and the ensemble feature selection using the union operation and the wrapper feature selection (REF). In this stage, the features used to construct the network flow from the used dataset are excluded to ensure that the DL models do not utilize them as patterns.

The features of the *f* set are equal to the number of features in the used dataset, and the features of f_e are as follows:

 

  f_e=[Label, Cat,Timestamp,Dst_Port,Protocol, Sub_Cat,Flow_ID,

Src_IP,Src_Port,Dst_IP]

 

The features selected by VT are denoted as F_vt, and the result is as follows:

 

  F_vt=[Fwd_Pkt_Len_Max, Fwd_Pkt_Len_Min, Fwd_Pkt_Len_Mean,

Bwd_Pkt_Len_Max, Bwd_Pkt_Len_Min, Bwd_Pkt_Len_Mean, Fwd_Pkts/s,

Bwd_Pkts/s, Pkt_Len_Min, RST_Flag_Cnt, ECE_Flag_Cnt,

Down/Up_Ratio, Pkt_Size_Avg]

 

The features selected by MI are denoted as F_mi, and the result is as follows:

 

  F_mi=[Flow_Duration, TotLen_Bwd_Pkts, Flow_Byts/s, Flow_IAT_Mean,

Bwd_Header_Len, RST_Flag_Cnt, Subflow_Fwd_Byts,

Subflow_Bwd_Byts, Active_Max, Idle_Mean]

 

The features selected by Chi are denoted as F_cs, and the result is as follows:

 

  F_cs=[Fwd_Pkt_Len_Max, Fwd_Pkt_Len_Mean, Fwd_Pkt_Len_Std,

Bwd_Pkt_Len_Min, Pkt_Len_Max, Pkt_Len_Mean, Pkt_Len_Var,

RST_Flag_Cnt, Down/Up_Ratio, Subflow_Bwd_Byts]

 

The features selected by ANOVA are denoted as F_an, and the result is as follows:

 

  F_an=[TotLen_Fwd_Pkts, Fwd_Pkt_Len_Max, Fwd_Pkt_Len_Std,

Fwd_URG_Flags, Bwd_URG_Flags, Pkt_Len_Max, Pkt_Len_Mean,

RST_Flag_Cnt, Bwd_Blk_Rate_Avg, Subflow_Bwd_Byts]

 

The features selected by L1-based are denoted as F_l1, and the result is as follows:

 

  F_l1=[Fwd_Pkt_Len_Max, Bwd_Pkt_Len_Max, Bwd_Pkt_Len_Min,

Bwd_Pkt_Len_Mean, Fwd_Pkts/s, Bwd_Pkts/s, Pkt_Len_Min,

RST_Flag_Cnt, ECE_Flag_Cnt, Pkt_Size_Avg]

 

The results of F_vt, F_mi, F_cs, F_an, and F_l1 are combined using the union operation. The resulting set of features is denoted as F_u, and the result is as follows:

 

  F_u=[Fwd_Pkt_Len_Max, Bwd_Pkt_Len_Min, Idle_Mean, Down/Up_Ratio,

ECE_Flag_Cnt, Subflow_Bwd_Byts, Fwd_Pkt_Len_Std, TotLen_Bwd_Pkts,

Bwd_Blk_Rate_Avg, Bwd_URG_Flags, Active_Max, Bwd_Header_Len,

Bwd_Pkts/s, Pkt_Len_Var, Pkt_Len_Mean, Pkt_Len_Min, RST_Flag_Cnt,

Flow_IAT_Mean,Fwd_Pkts/s, Subflow_Fwd_Byts, Fwd_URG_Flags,

TotLen_Fwd_Pkts,Flow_Byts/s, Pkt_Len_Max, Pkt_Size_Avg,

Fwd_Pkt_Len_Mean, Bwd_Pkt_Len_Max,Flow_Duration,Bwd_Pkt_Len_Mean,

Fwd_Pkt_Len_Min]

 

The union operation aims to select unique features from various subsets obtained through filter feature selection methods. However, it overlooks the internal redundancy or irrelevance of features concerning prediction information, which can potentially result in an increased number of selected features (e.g., the number of features resulting from a union operation increases from 10 to 30). Thus, the feature set selected by the operation is fed to REF. The features selected by REF are denoted as F_REF, and the result is as follows:

 

  F_REF = [Fwd_Pkt_Len_Max, Idle_Mean, Fwd_Pkt_Len_Std, Bwd_Header_Len,

Bwd_Pkts/s,Flow_IAT_Mean, Pkt_Len_Max, Pkt_Size_Avg, Bwd_Pkt_Len_Max,

Flow_Duration]

 

The features in the $F_{R}EF$ feature set are considered significant and used as input for the next stage. The rationale for feature selection and the importance and relevance of the selected features to IoT attack detection is provided in Table 4.

### 5.4. The Performance of Various DL Models

The quality of the features utilized to train DL models significantly impacts their final performance. Selecting suitable and relevant characteristics becomes vital to guaranteeing the efficacy and accuracy of DL models. Selecting appropriate features allows DL models to boost their effectiveness in classification, prediction, and anomaly detection. When DL models are given poor quality or irrelevant features, they may struggle to acquire meaningful representations, leading to subpar performance or even a failure to converge. Therefore, the employed DL models, CNN, RNN, LSTM, and GRU, are trained based on the F_REF feature set. The parameters used in DL models are listed in Table 5. The architecture of RNN, LSTM, and GRU is the same as in ref. [42], while the architecture of CNN is the same as in ref. [43]. The performance of various DL models based on features selected by VT, MI, Chi-square, ANOVA, L1-based, union operation, and REF is tabulated in Table 6, Table 7, Table 8, Table 9, Table 10, Table 11 and Table 12.

**Figure 2:**
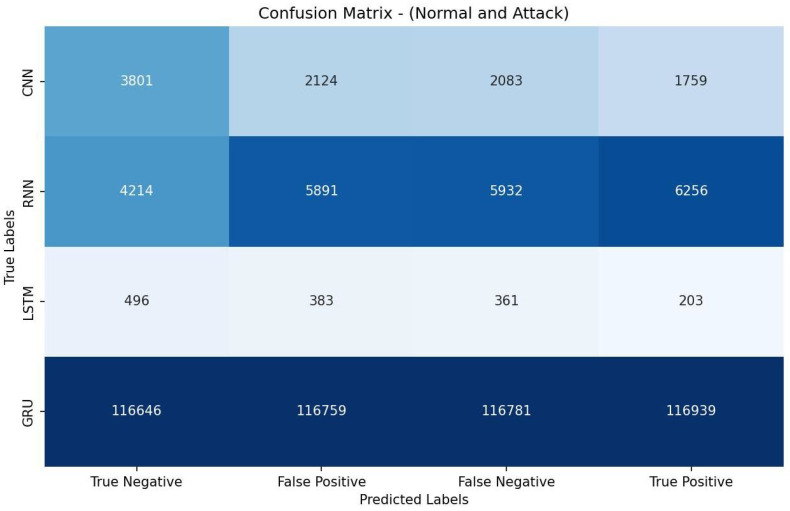
Confusion matrix of DL models based on features selected by VT.

Table 6 shows that the CNN achieved the highest accuracy of 96.24% and also demonstrated the best precision (96.51%), recall score (99.58%), and F1 measure (98.02%). It exhibited a higher AUC-ROC value (73.50% ) than the other models. These results indicate that the CNN model was most effective for the given detection task. The evaluation metrics in Table 6 are calculated based on the confusion matrix depicted in Figure 2.

**Figure 3:**
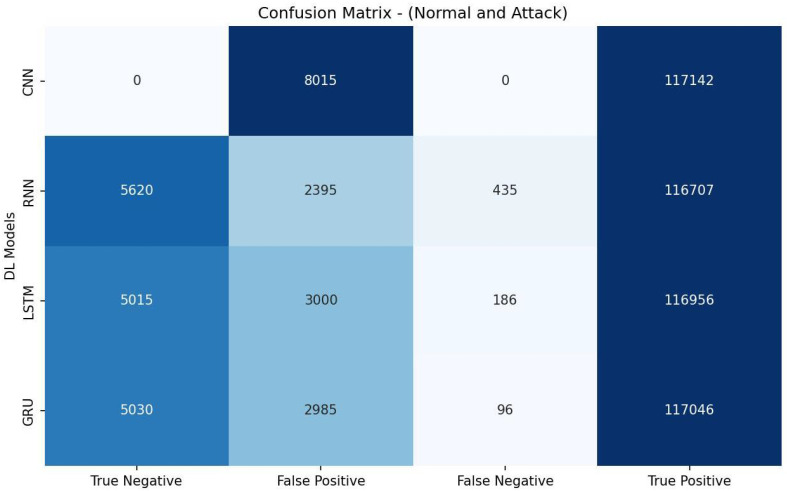
Confusion matrix of DL models based on features selected by MI.

Table 7 shows that the RNN model achieved the highest accuracy of 97.74% and demonstrated the best precision (97.99%), recall score (99.63%), and F1 measure (98.80%). It also had the highest AUC-ROC value (84.87%). These results indicate that the RNN model performed the most effectively for the given detection task, outperforming the other models (CNN, LSTM, and GRU) regarding overall detection accuracy. The evaluation metrics listed in Table 7 are calculated based on the confusion matrix depicted in Figure 3.

**Figure 4:**
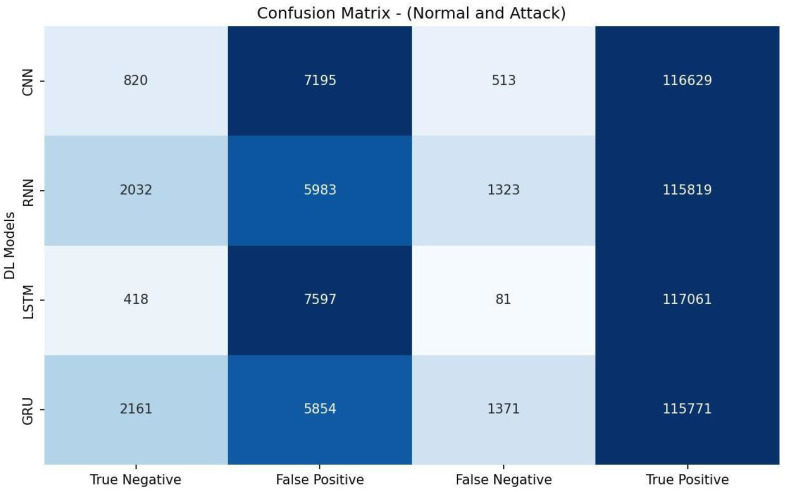
Confusion matrix of DL models based on features selected by Chi-square.

Table 8 shows that the RNN model achieved the highest accuracy of 94.16% and demonstrated the best precision (95.09%), recall score (98.87%), and F1 measure (96.94%). The RNN model also had a relatively high AUC-ROC value of 62.11%. These results suggest that the RNN model performed the most effectively for the given detection task, making it the top-performing model among the evaluated CNN, LSTM, and GRU models. The evaluation metrics listed in Table 8 are calculated based on the confusion matrix depicted in Figure 4.

**Figure 5:**
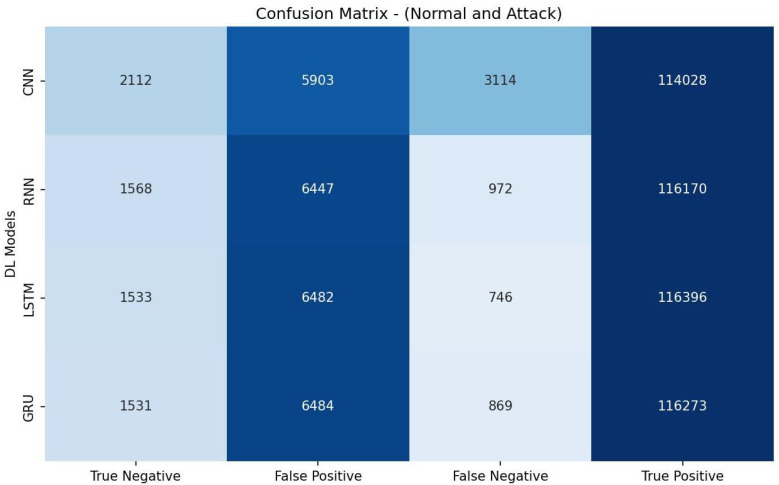
Confusion matrix of DL models based on features selected by Anova.

Table 9 shows that the LSTM model achieved the highest accuracy of 94.23% and demonstrated the best precision (94.73%), recall score (99.36%), and F1 measure (96.99%). It also had a competitive AUC-ROC value of 59.25%. The evaluation metrics listed in Table 9 are calculated based on the confusion matrix depicted in Figure 5.

The RNN model achieved an accuracy of 94.07%, with precision, recall score, and F1 measure values of 94.74%, 99.17%, and 96.91%, respectively. However, it had a slightly lower AUC-ROC of 59.37%.

The CNN model achieved an accuracy of 92.80%, with precision, recall score, and F1 measure values of 95.08%, 97.34%, and 96.20%, respectively. It had an AUC-ROC of 61.85%.

The GRU model achieved an accuracy of 94.13%, with precision, recall score, and F1 measure values of 94.72%, 99.26%, and 96.94%, respectively. It had an AUC-ROC of 59.18%.

Considering the metrics, the LSTM model performed the best among the evaluated deep learning models, achieving the highest accuracy and exhibiting vital precision, recall score, and F1 measure, making it the most effective model for the given detection task.

**Figure 6:**
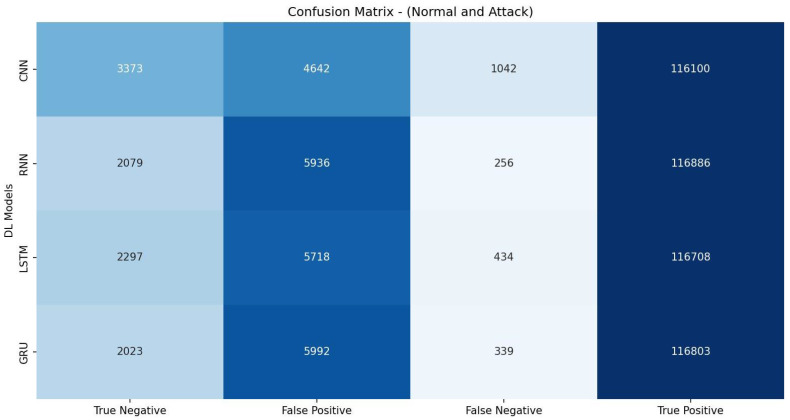
Confusion matrix of DL models based on features selected by L1-based.

Table 10 shows that the CNN model achieved the highest accuracy of 95.46% and demonstrated the best precision (96.16%), recall score (99.11%), and F1 measure (97.61%). It had an AUC-ROC value of 70.60%. The evaluation metrics listed in Table 10 are calculated based on the confusion matrix depicted in Figure 6.

The RNN model achieved an accuracy of 95.05%, with precision, recall score, and F1 measure values of 95.17%, 99.78%, and 97.42%, respectively. It had an AUC-ROC of 62.86%.

The LSTM model achieved an accuracy of 95.09%, with precision, recall score, and F1 measure values of 95.33%, 99.63%, and 97.43%, respectively. It had an AUC-ROC of 64.14%.

The GRU model achieved an accuracy of 94.94%, with precision, recall score, and F1 measure values of 95.12%, 99.71%, and 97.36%, respectively. It had an AUC-ROC of 62.48%.

The CNN model outperformed the evaluated deep learning models when assessing the metrics. It attained the highest accuracy and exhibited impressive precision, recall score, and F1 measure.

**Figure 7:**
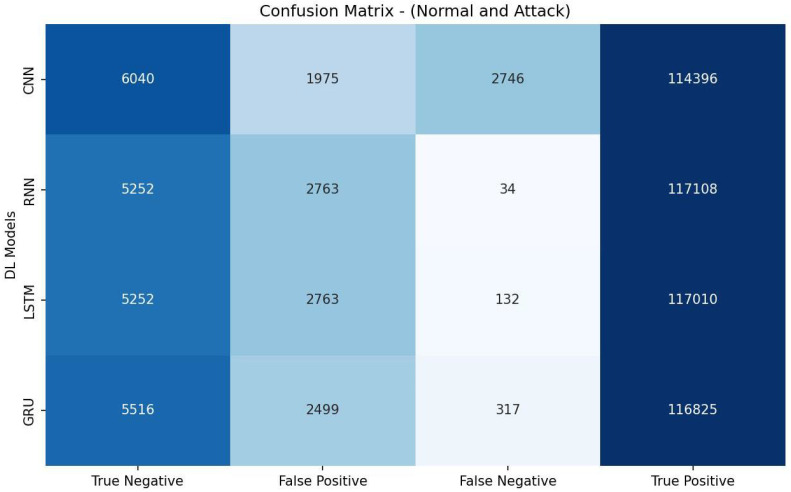
Confusion matrix of DL models based on features selected by union operation.

The union operation is the first ensemble method applied to the selected five filter feature techniques. Table 11 shows that the CNN model achieved an accuracy of 96.23%, demonstrating high precision (98.30%) and recall score (97.66%). It also had a good F1 measure of 97.98%. However, it had an AUC-ROC value of 86.51%. The evaluation metrics listed in Table 11 are calculated based on the confusion matrix depicted in Figure 7.

The RNN model achieved the highest accuracy of 97.77% among the models. It had a precision of 97.70% and an impressive recall score of 99.97%. The F1 measure for the RNN model was 98.82%, indicating a good balance between precision and recall. It had an AUC-ROC of 82.75%.

The LSTM model achieved an accuracy of 97.69% with a precision and recall score of 97.69% and 99.89%, respectively. The F1 measure for LSTM was 98.78%, with an AUC-ROC value of 82.71

The GRU model achieved an accuracy of 97.75% with a precision of 97.91% and a recall score of 99.73%. The F1 measure for GRU was 98.81%. However, it had a higher AUC-ROC of 84.2%.

The RNN model demonstrated the best performance, achieving the highest accuracy and recall score. The CNN and LSTM models also performed well.

**Figure 8:**
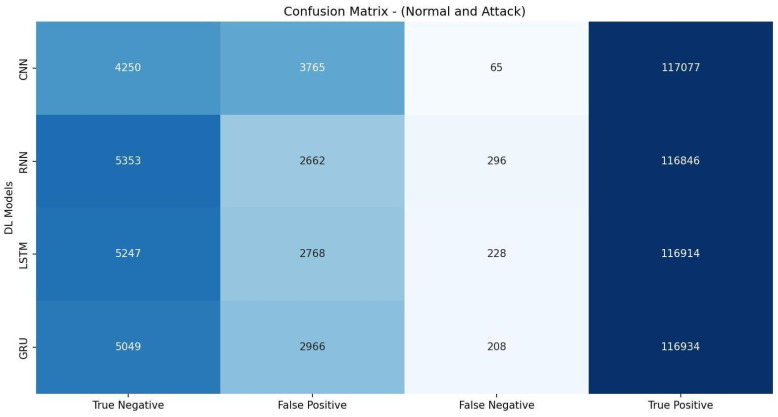
Confusion matrix of DL models based on features selected by REF.

Table 12 shows that the CNN model achieved an accuracy of 97.05%, with a precision of 96.99% and a high recall score of 99.95%. The F1 measure for the CNN model was 98.45%, indicating a good balance between precision and recall. It had an AUC-ROC value of 77.35%. The evaluation metrics listed in Table 12 are calculated based on the confusion matrix depicted in Figure 8.

The RNN model demonstrated an accuracy of 97.80%, with a precision of 97.86% and a recall score of 99.84%. The F1 measure for the RNN model was 98.84%, indicating a good balance between precision and recall. It had an AUC-ROC of 83.93%.

The LSTM model achieved an accuracy of 97.86%, with a precision of 97.95% and a recall score of 99.80%. The F1 measure for LSTM was 98.87%, indicating a good balance between precision and recall. It had an AUC-ROC value of 84.67%.

The GRU model achieved an accuracy of 97.87%, with a precision of 97.90% and a recall score of 99.87%. The F1 measure for GRU was 98.87%. It had an AUC-ROC of 84.25%.

All the DL models performed well, achieving high accuracy and recall scores. The LSTM and GRU models demonstrated slightly higher precision than the CNN and RNN models. The LSTM model had the highest AUC-ROC value, indicating its effectiveness in classification.

### 5.5. Discussion

The results presented in Table 6, Table 7, Table 8, Table 9 and Table 10 demonstrate that using a single feature selection technique alone had a negative effect on the performance of DL models. This implies that relying on a single technique to select features for the models resulted in sub-optimal performance. Table 11, on the other hand, demonstrates that employing the union operation on the feature sets selected by filter feature selection techniques improved the performance of DL models compared with using a single feature selection technique. Combining the selected features from multiple filter techniques enhanced the model’s performance. Additionally, Table 12 indicates that wrapper feature selection REF further improved the model’s performance. Table 13 shows the improvement of REF over the union operation.

Based on Table 13, REF exhibited substantial enhancements over the union operation for the CNN model (0.82 improvements), while the improvements for RNN, LSTM, and GRU models were smaller (0.03, 0.17, and 0.12, respectively).

For Precision, REF showed significant improvements over the union operation for the CNN (0.69 improvements) and LSTM (0.26 improvements) models, whereas the improvements for RNN and GRU models were moderate (0.16 and 0.01, respectively).

Regarding Recall Score, the REF technique resulted in substantial improvements for the CNN model (2.29 improvement), while the improvements for RNN, LSTM, and GRU models were minor (0.13, 0.09, and 0.14, respectively).

The F1 Measure showed a decrease for the CNN model (−9.16) when using REF compared with the union operation. However, there were slight improvements for the RNN (0.02 improvement), LSTM (0.09 improvement), and GRU (0.06 improvement) models.

Regarding the AUC-ROC metric, the CNN model experienced a significant decrease (−9.16) when using REF, whereas the RNN (1.18 improvement), LSTM (0.96 improvement), and GRU (0.03 improvement) models showed moderate improvements.

Furthermore, we have conducted a comparison between our proposed approach and the approach presented in [30]. This comparison is based on using the same dataset, implementing feature selection, and using DL algorithms as classifiers, ensuring a fair assessment. Additionally, we implemented the DNN model used in ref. [30]. The top ten features selected in their work are listed below:

 

*F*′ = [*Src_Port*, *Bwd_Pkts/s*, *Dst_Port*, *Flow_IAT_Mean*, *Flow_Duration*,

*Flow_Pkts/s*, *Bwd_IAT_Tot*, *Pkt_Size_Avg*, *Pkt_Len_Mean*, *Bwd_IAT_Max*,

*ACK_Flag_Cnt*, *Flow_IAT_Max*]

 

Table 14 shows the impact of the F_REF selected by the proposed approach and $F{}^{\prime }$ on the performance of the DNN model.

As shown in Table 14, the proposed approach (F_REF+DNN) demonstrates superior performance across all evaluated metrics when compared with the approach in ref. [30]. This indicates the effectiveness of the proposed ensemble feature selection mechanism in selecting discriminative features from various feature selection techniques, contributing to enhancing the performance of IDS.

In contrast, the work proposed in ref. [30] relies on a single feature selection method that may be unable to select significant features. In our proposed approach, the features $Src_{P}ort$ and $Dst_{P}ort$ are eliminated, as they might introduce patterns that could influence the DL detection process, as explained in Section 4.2. However, these features have been used in ref. [30].

In summary, the experimental results indicate that the proposed ensemble feature selection technique has significantly enhanced the performance of the CNN, RNN, LSTM, GRU, and DNN models. The proposed ensemble feature selection technique has likely improved the models’ ability to select relevant features, resulting in better overall performance.

## 6. Conclusions and Future Works

This paper proposes an approach for detecting attacks on IoT networks by utilizing ensemble feature selection and DL models. The ensemble feature selection process combines the outputs of five different filter selection techniques: variance threshold, mutual information, Chi-square, ANOVA, and L1-based methods. These techniques are applied individually, and their outputs are combined through a union operation. To refine the feature selection and avoid potential issues related to redundancy and irrelevance, we employ a wrapper algorithm called RFE to select the top ten best features from the union operation output. Experiments on the IoT-Botnet 2020 dataset evaluated the impact of the selected feature set on DL models’ performance. All evaluated DL models achieved high values for detection accuracy (ranging between 97.05–97.87%), precision (ranging between 96.99–97.95%), recall (ranging between 99.80–99.95%), and F1 measure (ranging between 98.45–97.87%). Additionally, CNN exhibited the lowest AUC-ROC value, with a value of 77.35%. For future work, we plan to extend our investigation and explore the impact of different feature selection methods on DL models’ performance in detecting IoT network attacks. This will involve exploring a wider range of feature selection techniques beyond our current approach. Incorporating alternative wrapper feature selection algorithms, such as Genetic Algorithms, Forward-Backward Search, and Sequential Feature Selection, may yield different results, allowing us to assess their effectiveness compared with RFE.

## Figures and Tables

**Table 1:** IoT Attacks vs. Traditional Attacks.

Aspect	IoT Attacks	Traditional Attacks
Target	IoT devices and systems	Traditional computing devices and networks
Attack Vector	Exploiting vulnerabilities in IoT systems	Exploiting software vulnerabilities
Scale and Scope	Can target large-scale IoT networks	Primarily focused on individual devices
Consequences	Privacy breaches, data theft, physical harm	Data breaches, financial losses, system crashes
Attack Types	Botnet attacks, unauthorized access and control, firmware vulnerabilities	Malware, phishing, ransomware, SQL injection
Security Challenges	Device heterogeneity, lack of standard protocols, weak authentication	Vulnerabilities in software, social engineering
Potential Impacts	Disruption of critical services, financial losses, compromised privacy	Data breaches, unauthorized access, system downtime
Mitigation Strategies	Strong authentication, encryption, regular updates, network segmentation	Patch management, IDS

**Table 2:** Summary of existing research works.

Ref.	Approach	Dataset	Key Findings
[19]	System-level IDS using behavior profiling	3973 IoT malware samples	Detection efficiency ranging from 78% to 100%
[20]	DDoS botnet IDS based on deep learning	Realistic network environment	Outperformed state-of-the-art systems in accuracy
[21]	Feature selection for DoS and DDoS IDS	IoT-BoT and KDD Cup 1999 datasets	Superior performance with reduced number of features
[22]	Ensemble tree-based IDS with ML models	NF-BoT-IoT-v2, NF-ToN-IoT-v2, and IoTDS20 datasets	Improved attack detection efficiency
[23]	DNN-based IDS for IoMT networks	Networks of IoMT	Improved accuracy and faster detection
[24]	Deep learning approach for botnet detection	Network traffic flow	Reliable data for effective IoT applications
[25]	Deep learning model for anomaly detection in IoT networks	IoT networks	Improved F1-score, accuracy, precision, and recall
[26]	CNN-based IDS for IoT security	BoT-IoT datasets	Achieved an accuracy of 92.85%in detecting intrusions
[27]	Deep hierarchical network for analyzing malicious activity	Raw packet data	Successful detection of certain types of attacks
[28]	Deep learning-based IDS for medical IoT systems	Medical IoT devices	High accuracy in detecting attacks and anomalies
[29]	Deep migration learning model for IoT intrusion detection	KDD CUP 99	Outperformed existing algorithms in feature selection
[30]	Approach using MI and DNN for IoT attack detection	IoT-Botnet 2020	Outperformed existing algorithms in feature selection and detection accuracy

**Table 3:** Records distribution of IoT-Botnet 2020.

Category	Value
No. of normal category records	40,073
No. of attack category records	585,710
Total No. of records	625,783
No. of categorical features	6
No. of numerical features	79
Total no. of features	85

**Table 4:** Rationale of selected features and their importance and relevance to IoT attacks.

Feature	Rational of Selection
Fwd_Pkt_Len_Max	It’s crucial for spotting huge packets, which might point to malicious data transfers or attacks aiming to overload the IoT network.
Idle_Mean	Abnormally low amounts of time spent doing nothing may point to hostile communication patterns, which in turn could indicate malicious intent.
Bwd_Header_Len	is useful for detecting abnormally large header lengths, which may point to exploit attempts or other suspicious network activity.
Bwd_Pkts/s	Abnormally high rates might indicate an effort to overwhelm the network or a flooding attack.
Flow_IAT_Mean	Abnormal changes in the average time between arrivals might point to illicit activity.
Pkt_Len_Max	The maximum packet length in a flow is useful for detecting out-of-the-ordinary packet sizes that might indicate an effort to circumvent security controls.
Pkt_Size_Avg	The average size of packets in a flow reveals common data transfer behaviors throughout the network. Deviations from the average might be indicative of potential attacks.
Bwd_Pkt_Len_Max	is a feature that, like the maximum packet length feature before it, analyzes backward packets to provide information about possible attacks coming from the opposite way.
Flow_Duration	Flows that last for a long time might be an indication of extensive communication, such as when a huge amount of data is being sent or when an attack is being attempted.

**Table 5:** The parameters used in DL models.

Parameter	Description
Loss function	Sparse categorical cross-entropy
Optimizer	Adam
Learning rate	0.01
Early stopping	Terminated if validation loss is not reduced after three iterations
Epoch count	100

**Table 6:** The performance of various DL models based on features selected by VT.

DL Model	Detection Accuracy (%)	Precision (%)	Recall Score (%)	F1 Measure (%)	AUC-ROC (%)
CNN	96.24	96.51	99.58	98.02	73.50
RNN	94.99	95.20	99.67	97.38	63.09
LSTM	94.97	95.17	99.69	97.38	62.84
GRU	94.84	94.92	99.83	97.31	60.89

**Table 7:** The performance of various DL models based on features selected by MI.

DL Model	Detection Accuracy (%)	Precision (%)	Recall Score (%)	F1 Measure (%)	AUC-ROC (%)
CNN	93.60	93.60	100.00	96.69	50.00
RNN	97.74	97.99	99.63	98.80	84.87
LSTM	97.45	97.50	99.84	98.66	81.21
GRU	97.54	97.51	99.92	98.70	81.34

**Table 8:** The performance of various DL models based on features selected by Chi-square.

DL Model	Detection Accuracy (%)	Precision (%)	Recall Score (%)	F1 Measure (%)	AUC-ROC (%)
CNN	93.84	94.19	99.56	96.80	54.90
RNN	94.16	95.09	98.87	96.94	62.11
LSTM	93.87	93.91	99.93	96.83	52.57
GRU	94.23	95.19	98.83	96.97	62.90

**Table 9:** The performance of various DL models based on features selected by Anova.

DL Model	Detection Accuracy (%)	Precision (%)	Recall Score (%)	F1 Measure (%)	AUC-ROC (%)
CNN	92.80	95.08	97.34	96.20	61.85
RNN	94.07	94.74	99.17	96.91	59.37
LSTM	94.23	94.73	99.36	96.99	59.25
GRU	94.13	94.72	99.26	96.94	59.18

**Table 10:** The performance of various DL models based on features selected by L1-based.

DL Model	Detection Accuracy (%)	Precision (%)	Recall Score (%)	F1 Measure (%)	AUC-ROC (%)
CNN	95.46	96.16	99.11	97.61	70.60
RNN	95.05	95.17	99.78	97.42	62.86
LSTM	95.09	95.33	99.63	97.43	64.14
GRU	94.94	95.12	99.71	97.36	62.48

**Table 11:** The performance of various DL models based on union operation.

DL Model	Detection Accuracy (%)	Precision (%)	Recall Score (%)	F1 Measure (%)	AUC-ROC (%)
CNN	96.23	98.30	97.66	97.98	86.51
RNN	97.77	97.70	99.97	98.82	82.75
LSTM	97.69	97.69	99.89	98.78	82.71
GRU	97.75	97.91	99.73	98.81	84.28

**Table 12:** The performance of various DL models based on features selected by REF.

DL Model	Detection Accuracy (%)	Precision (%)	Recall Score (%)	F1 Measure (%)	AUC-ROC (%)
CNN	97.05	96.99	99.95	98.45	77.35
RNN	97.80	97.86	99.84	98.84	83.93
LSTM	97.86	97.95	99.80	98.87	84.67
GRU	97.87	97.90	99.87	98.87	84.25

**Table 13:** The improvement of REF over the union operation.

DL Model	Accuracy Improvement	Precision Improvement	Recall Score Improvement	F1 Measure Improvement	AUC-ROC Improvement
CNN	0.82	0.69	2.29	0.47	−9.16
RNN	0.03	0.16	0.13	0.02	1.18
LSTM	0.17	0.26	0.09	0.09	0.96
GRU	0.12	0.01	0.14	0.06	0.03

**Table 14:** Impact of F_REF and $F{}^{\prime }$ on the performance DNN model.

Metric	Proposed Approach F_REF + DNN	Approach in ref. [30] $F{}^{\prime }$ + DNN
TN	4786	0
FP	3229	8015
FN	57	0
TP	11,708	117,142
Detection Accuracy	97.37%	93.60%
Precision	97.32%	93.60%
Recall	99.95%	100.00%
F1 Score	98.62%	96.69%
AUC-ROC	79.83%	50.00%

## Data Availability

Not applicable.

## References

[1] Bahashwan A.A., Anbar M., Abdullah N., Al-Hadhrami T., Hanshi S.M., Saeed F., Al-Hadhrami T., Mohammed F., Mohammed E. (2021). Review on Common IoT Communication Technologies for Both Long-Range Network (LPWAN) and Short-Range Network. Proceedings of the Advances on Smart and Soft Computing.

[2] Internet of Threats: IoT Botnets Drive Surge in Network Attacks. https://securityintelligence.com/posts/internet-of-threats-iot-botnets-network-attacks/.

[3] Al-Amiedy T.A., Anbar M., Belaton B., Bahashwan A.A., Hasbullah I.H., Aladaileh M.A., Mukhaini G.A. (2023). A systematic literature review on attacks defense mechanisms in RPL-based 6LoWPAN of Internet of Things. Internet Things.

[4] Albulayhi K., Al-Haija Q.A., Alsuhibany S.A., Jillepalli A.A., Ashrafuzzaman M., Sheldon F.T. (2022). IoT Intrusion Detection Using Machine Learning with a Novel High Performing Feature Selection Method. Appl. Sci..

[5] Mehmod T., Rais H.B., Soh P., Woo W., Sulaiman H., Othman M., Saat M. (2016). Ant colony optimization and feature selection for intrusion detection. Advances in Machine Learning and Signal Processing.

[6] Asharf J., Moustafa N., Khurshid H., Debie E., Haider W., Wahab A. (2020). A review of intrusion detection systems using machine and deep learning in internet of things: Challenges, solutions and future directions. Electronics.

[7] Xenofontos C., Zografopoulos I., Konstantinou C., Jolfaei A., Khan M.K., Choo K.K.R. (2022). Consumer, Commercial, and Industrial IoT (In)Security: Attack Taxonomy and Case Studies. IEEE Internet Things J..

[8] Al-Ani A.K., Anbar M., Al-Ani A., Ibrahim D.R. (2020). Match-Prevention Technique Against Denial-of-Service Attack on Address Resolution and Duplicate Address Detection Processes in IPv6 Link-Local Network. IEEE Access.

[9] Alieyan K., Kadhum M.M., Anbar M., Rehman S.U., Alajmi N.K.A. An overview of DDoS attacks based on DNS. Proceedings of the 2016 International Conference on Information and Communication Technology Convergence (ICTC).

[10] Ahmed Z., Danish S.M., Qureshi H.K., Lestas M. Protecting IoTs from mirai botnet attacks using blockchains. Proceedings of the IEEE International Workshop on Computer Aided Modeling and Design of Communication Links and Networks, CAMAD.

[11] Hasan M.K., Ghazal T.M., Saeed R.A., Pandey B., Gohel H., Eshmawi A.A., Abdel-Khalek S., Alkhassawneh H.M. (2022). A review on security threats, vulnerabilities, and counter measures of 5G enabled Internet-of-Medical-Things. IET Commun..

[12] Alamiedy T.A., Anbar M., Al-Ani A.K., Al-Tamimi B.N., Faleh N. (2019). Review on feature selection algorithms for anomaly-based intrusion detection system. Advances in Intelligent Systems and Computing.

[13] Al Fatih Abil Fida M., Ahmad T., Ntahobari M. Variance Threshold as Early Screening to Boruta Feature Selection for Intrusion Detection System. Proceedings of the 2021 IEEE 13th International Conference on Information and Communication Technology and System, ICTS 2021.

[14] Gümüşbaş D., Yıldırım T., Genovese A., Scotti F. (2021). A comprehensive survey of databases and deep learning methods for cybersecurity and intrusion detection systems. IEEE Syst. J..

[15] Thaseen I.S., Kumar C.A., Ahmad A. (2019). Integrated Intrusion Detection Model Using Chi-Square Feature Selection and Ensemble of Classifiers. Arab. J. Sci. Eng..

[16] Brereton R.G. (2019). Introduction to analysis of variance. J. Chemom..

[17] Shekar B.H., Dagnew G. (2020). L1-Regulated Feature Selection and Classification of Microarray Cancer Data Using Deep Learning. Advances in Intelligent Systems and Computing.

[18] Mohammed B., Gbashi E. (2021). Intrusion Detection System for NSL-KDD Dataset Based on Deep Learning and Recursive Feature Elimination. Eng. Technol. J..

[19] Mudgerikar A., Sharma P., Bertino E. E-Spion: A system-level intrusion detection system for IoT devices. Proceedings of the AsiaCCS 2019—Proceedings of the 2019 ACM Asia Conference on Computer and Communications Security.

[20] Jithu P., Shareena J., Ramdas A., Haripriya A.P. (2021). Intrusion Detection System for IOT Botnet Attacks Using Deep Learning. SN Comput. Sci..

[21] Nimbalkar P., Kshirsagar D. (2021). Feature selection for intrusion detection system in Internet-of-Things (IoT). ICT Express.

[22] Le T.T.H., Kim H., Kang H., Kim H. (2022). Classification and Explanation for Intrusion Detection System Based on Ensemble Trees and SHAP Method. Sensors.

[23] Swarna Priya R.M., Maddikunta P.K.R., Parimala M., Koppu S., Gadekallu T.R., Chowdhary C.L., Alazab M. (2020). An effective feature engineering for DNN using hybrid PCA-GWO for intrusion detection in IoMT architecture. Comput. Commun..

[24] Sriram S., Vinayakumar R., Alazab M., Soman K.P. Network flow based IoT botnet attack detection using deep learning. Proceedings of the IEEE INFOCOM 2020—IEEE Conference on Computer Communications Workshops, INFOCOM WKSHPS 2020.

[25] Yin C., Zhang S., Wang J., Xiong N.N. (2022). Anomaly Detection Based on Convolutional Recurrent Autoencoder for IoT Time Series. IEEE Trans. Syst. Man Cybern. Syst..

[26] Saba T., Rehman A., Sadad T., Kolivand H., Bahaj S.A. (2022). Anomaly-based intrusion detection system for IoT networks through deep learning model. Comput. Electr. Eng..

[27] Wang B., Su Y., Zhang M., Nie J. (2020). A deep hierarchical network for packet-level malicious traffic detection. IEEE Access.

[28] Manimurugan S., Al-Mutairi S., Aborokbah M.M., Chilamkurti N., Ganesan S., Patan R. (2020). Effective attack detection in internet of medical things smart environment using a deep belief neural network. IEEE Access.

[29] Li D., Deng L., Lee M., Wang H. (2019). IoT data feature extraction and intrusion detection system for smart cities based on deep migration learning. Int. J. Inf. Manag..

[30] Ahmad Z., Khan A.S., Nisar K., Haider I., Hassan R., Haque M.R., Tarmizi S., Rodrigues J.J. (2021). Anomaly detection using deep neural network for iot architecture. Appl. Sci..

[31] Akhiat Y., Touchanti K., Zinedine A., Chahhou M. (2023). IDS-EFS: Ensemble feature selection-based method for intrusion detection system. Multimed. Tools Appl..

[32] Wu T., Hao Y., Yang B., Peng L. (2023). ECM-EFS: An ensemble feature selection based on enhanced co-association matrix. Pattern Recognit..

[33] Kim J., Kim J., Kim H., Shim M., Choi E. (2020). CNN-based network intrusion detection against denial-of-service attacks. Electronics.

[34] Sahu A.K., Sharma S., Tanveer M., Raja R. (2021). Internet of Things attack detection using hybrid Deep Learning Model. Comput. Commun..

[35] Pallasdies F., Norton P., Schleimer J.H., Schreiber S. (2021). Neural optimization: Understanding trade-offs with Pareto theory. Curr. Opin. Neurobiol..

[36] Ullah I., Mahmoud Q.H. A Technique for Generating a Botnet Dataset for Anomalous Activity Detection in IoT Networks. Proceedings of the Conference Proceedings—IEEE International Conference on Systems, Man and Cybernetics.

[37] Koroniotis N., Moustafa N., Sitnikova E., Turnbull B. (2019). Towards the development of realistic botnet dataset in the Internet of Things for network forensic analytics: Bot-IoT dataset. Future Gener. Comput. Syst..

[38] Deeb Al-Mo A.A., Wan T.C., Al-Saedi K., Altaher A., Ramadass S., Manasrah A., Melhiml L.B., Anbar M. (2011). An online model on evolving phishing e-mail detection and classification method. J. Appl. Sci..

[39] Inayat U., Zia M.F., Mahmood S., Khalid H.M., Benbouzid M. (2022). Learning-Based Methods for Cyber Attacks Detection in IoT Systems: Methods, Analysis, and Future Prospects. Electronics.

[40] Zhang J., Pan L., Han Q.L., Chen C., Wen S., Xiang Y. (2022). Deep Learning Based Attack Detection for Cyber-Physical System Cybersecurity: A Survey. IEEE/CAA J. Autom. Sin..

[41] Rathore M.M., Saeed F., Rehman A., Paul A., Daniel A. Intrusion Detection using Decision Tree Model in High-Speed Environment. Proceedings of the ICSNS 2018—Proceedings of IEEE International Conference on Soft-Computing and Network Security.

[42] Elejla O.E., Anbar M., Hamouda S., Faisal S., Bahashwan A.A., Hasbullah I.H. (2022). Deep-Learning-Based Approach to Detect ICMPv6 Flooding DDoS Attacks on IPv6 Networks. Appl. Sci..

[43] Alabsi B.A., Anbar M., Rihan S.D.A. (2023). CNN-CNN: Dual Convolutional Neural Network Approach for Feature Selection and Attack Detection on Internet of Things Networks. Sensors.

